# Identification of an immune-regulated phagosomal Rab cascade in macrophages

**DOI:** 10.1242/jcs.144923

**Published:** 2014-05-01

**Authors:** Gang Pei, Urska Repnik, Gareth Griffiths, Maximiliano Gabriel Gutierrez

**Affiliations:** 1 Research Group Phagosome Biology, Helmholtz Centre for Infection Research, Inhoffenstrasse 7, 38124 Braunschweig, Germany; 2 Department of Biosciences, University of Oslo, Blindernveien 31, 0371 Oslo, Norway; 3 Division of Mycobacterial Research, Medical Research Council, National Institute for Medical Research, The Ridgeway, London NW7 1AA, UK

**Keywords:** Macrophage, Rab, Phagosome, Rabex-5, Lysosome

## Abstract

Interferon-γ (IFN-γ) has been shown to regulate phagosome trafficking and function in macrophages, but the molecular mechanisms involved are poorly understood. Here, we identify Rab20 as part of the machinery by which IFN-γ controls phagosome maturation. We found that IFN-γ stimulates the association of Rab20 with early phagosomes in macrophages. By using imaging of single phagosomes in live cells, we found that Rab20 induces an early delay in phagosome maturation and extends the time for which Rab5a and phosphatidylinositol 3-phosphate (PI3P) remain associated with phagosomes. Moreover, Rab20 depletion in macrophages abrogates the delay in phagosome maturation induced by IFN-γ. Finally, we demonstrate that Rab20 interacts with the Rab5a guanine nucleotide exchange factor Rabex-5 (also known as RABGEF1) and that Rab20 knockdown impairs the IFN-γ-dependent recruitment of Rabex-5 and Rab5a into phagosomes. Taken together, here, we uncover Rab20 as a key player in the Rab cascade by which IFN-γ induces a delay in phagosome maturation in macrophages.

## INTRODUCTION

Phagosomes are crucial components of the innate and adaptive immune systems (Jutras and Desjardins, 2005). The process known as ‘phagosome maturation’ involves a series of coordinated fusion events that lead to biochemical changes in the composition of a phagosome. Multiple intracellular compartments contribute to this pathway through dynamic events of fusion and cargo delivery. Thus, the entire machinery that regulates phagosome maturation is strongly linked to proteins that organize intracellular trafficking, such as Rab proteins and soluble N-ethylmaleimide-sensitive factor adaptor proteins receptors (SNAREs) (Fairn and Grinstein, 2012).

It is well established that cytokines modulate the outcome of phagosome maturation in myelocytic cells (Balce et al., 2011; Bhattacharya et al., 2006; Harris et al., 2007; O'Leary et al., 2011). Interferon- γ (IFN-γ) is a key cytokine that plays multiple roles during the activation and modulation of intracellular trafficking, especially in macrophages (Gordon, 2003; Montaner et al., 1999; Schroder et al., 2004). IFN-γ endows macrophages with the ability to control the immune function of phagosomes by stimulating phagosome maturation (Nathan et al., 1983; Santic et al., 2005a; Santic et al., 2005b; Schaible et al., 1998; Via et al., 1998). However, in the same cells, IFN-γ also delays phagosome maturation and acidification at early time points (Jutras et al., 2008; Trost et al., 2009; Tsang et al., 2000; Yates et al., 2007). This transient delay observed in macrophages is important for the adaptive immune response, because the slow degradation of peptide antigens is crucial for proper antigen cross-presentation in macrophages (Jutras et al., 2008; Trost et al., 2009). Although much is known about the role of IFN-γ in phagosome biology, little is known about the molecular machinery by which IFN-γ modulates phagosome trafficking and function.

Regulators of intracellular trafficking represent a potential molecular link between the activation of immune cells by cytokines and phagosome transport (Pei et al., 2012). Rab proteins are small GTPases that play a central role in phagosomal trafficking as well as in fusion between phagosomes and various compartments (Fairn and Grinstein, 2012). Moreover, the expression of multiple Rab proteins is modulated during the activation of macrophages, making Rab GTPases good candidates for mediating the cytokine control of phagosome maturation (Pei et al., 2012). Although >20 Rab proteins are associated with phagosomes, a defined role in phagosome biology is only known for a few of them (Li et al., 2010). In this context, the presence of immune-regulated Rab cascades that control cytokine-dependent phagosomal function could represent an attractive mechanism of regulation as shown for endocytosis and exocytosis (Hutagalung and Novick, 2011; Jean and Kiger, 2012; Pfeffer, 2013).

Among the Rab proteins, Rab20 is an especially promising candidate for being a regulator of an IFN-γ-dependent mediator of phagosome trafficking. Rab20 was originally described as being mainly associated with endocytic organelles (Lütcke et al., 1994). This small GTPase was proposed to regulate the trafficking of the vacuolar ATPase (vATPase) and connexin 43 (Cnx43, also known as GJA1) (Curtis and Gluck, 2005; Das Sarma et al., 2008). Proteomic studies have shown that Rab20 is recruited to phagosomes in IFN-γ-treated macrophages (Trost et al., 2009). In resting macrophages, overexpressed Rab20 associates with phagosomes, and the expression of the dominant-negative mutant of Rab20 impairs phagosome maturation (Egami and Araki, 2012; Seto et al., 2011). Consistent with a central role of Rab20 in immunity, the expression of this GTPase is highly upregulated after infection with different bacterial pathogens in multiple systems (Berry et al., 2010; Goldmann et al., 2007; Gutierrez et al., 2008; Mahapatra et al., 2010; Tailleux et al., 2008; van der Sar et al., 2009). However, a functional role for this GTPase in immunity remains to be identified.

Here, we investigated the role of Rab20 in the context of phagosome maturation and its modulation by IFN-γ. We found that IFN-γ enhanced the association of Rab20 with phagosomes in macrophages. This recruitment of Rab20 into phagosomes induced an early and transient delay in phagosome maturation. By contrast, either the expression of dominant-negative Rab20 or Rab20 depletion accelerated phagosome maturation and proteolytic activity, prematurely disassociating Rab5a from phagosomes. We show that the guanine nucleotide exchange factor (GEF) of Rab5a, Rabex-5 (also known as RABGEF1) is also an effector of Rab20, and the recruitment of Rab20 to phagosomes through IFN-γ signaling increased the association of Rabex-5 with phagosomes. Additionally, Rab20 depletion impaired the association of both Rabex-5 and Rab5a with phagosomes. Collectively, our data reveal a newly identified Rab cascade in phagosomes, in which IFN-γ induces Rab20-dependent recruitment of Rabex-5, which in turn transiently increases the recruitment of Rab5a to phagosomes, resulting in a delay in phagosome maturation.

## RESULTS

### Early association of Rab20 with phagosomes is enhanced by IFN-γ in macrophages

**Fig. 1.**
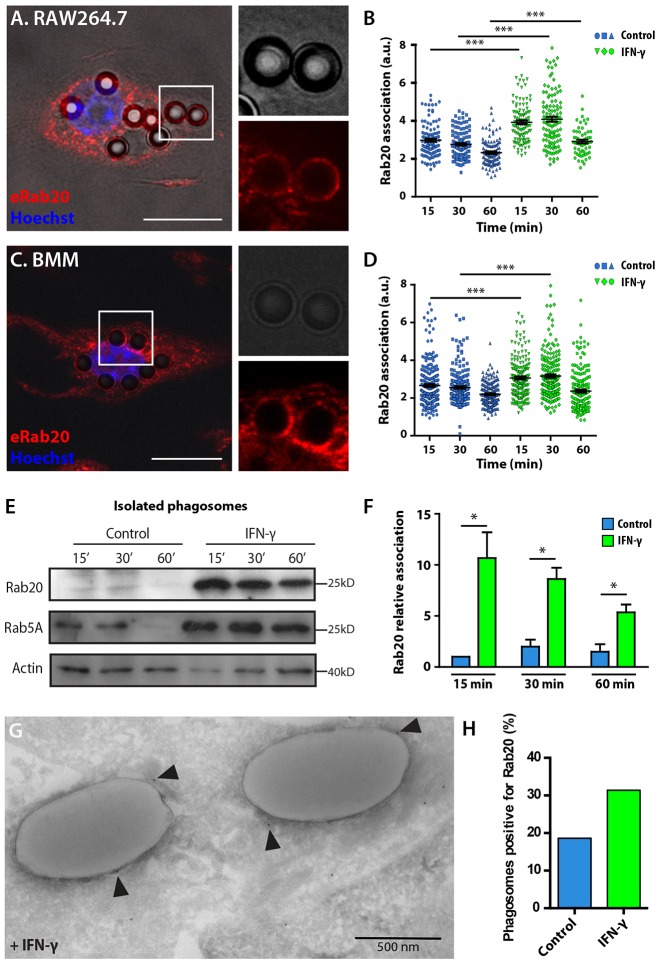
**Rab20 association with early phagosomes in IFN-γ-activated macrophages.** (A) Analysis of the association of endogenous Rab20 with phagosomes in RAW264.7 macrophages. RAW264.7 macrophages were incubated with 3-µm IgG-coated beads for 1 h and 15 min of chasing, and were subsequently fixed and stained for Rab20. Nuclei were stained with Hoechst 33258 and are shown in blue. Magnified images show the association of Rab20 with phagosomes. Scale bar: 10 µm. (B) Quantitative analysis of the association of endogenous Rab20 with phagosomes at the indicated time-points. Data show the mean±s.e.m. of one representative experiment out of three independent experiments. At least 100 phagosomes were analyzed in each experiment. ***P≤0.001 (Student's two-tailed unpaired *t*-test). (C) Analysis of the association of endogenous Rab20 with phagosomes in bone marrow macrophages (BMM) as in A. Scale bar: 10 µm. (D) Quantitative analysis of the association of endogenous Rab20 with phagosomes in BMM as in B. (E) Western blot analysis of the association of endogenous Rab20 with phagosomes in RAW264.7 macrophages that were untreated or treated with 200 U/ml IFN-γ for 16 h. Latex-bead phagosomes were isolated from untreated or IFN-γ-treated RAW264.7 macrophages after 15, 30 and 60 min of chase. Rab20 and Rab5a levels were detected and actin was used as the loading control. (F) Quantification of the association of Rab20 with phagosomes from E, calculated relative to actin and normalized to the value of untreated macrophages at 15 min. Data show the mean±s.e.m. of three independent experiments. *P≤0.05 (Mann-Whitney test). (G) IFN-γ-treated RAW264.7 macrophages were incubated with 1-µm IgG-coated beads for 1 h and then processed for cryosectioning. Rab20 was detected by using a rabbit anti-Rab20 antibody followed by labeling with 10-nm Protein-A–gold. Arrowheads indicate the presence of Rab20 at the phagosomal membrane. (H) Quantitative analysis of the number of phagosomes positive for Rab20 in cells treated with IFN-γ or left untreated.

Endogenous Rab20 was recruited to phagosomes in RAW264.7 and primary bone marrow macrophages (BMM, Fig. 1A,C). A quantitative analysis of the association of Rab20 with phagosomes indicated that the levels of association decreased from 15 min to 60 min. Remarkably, the association of Rab20 with phagosomes was significantly higher after treatment with IFN-γ (Fig. 1B,D). In dynamic studies, by using live-cell imaging of macrophages expressing EGFP–Rab20, it was possible to precisely determine that EGFP–Rab20 was recruited into phagosomes after actin dissociation (supplementary material Fig. S1A,B) and remained associated with early phagosomes for ∼20 min (supplementary material Fig. S1C,D), suggesting a function for Rab20 in early phagosomes. To confirm this hypothesis, IgG-coated latex-bead phagosomes were purified at different time-points from macrophages (that were either treated with IFN-γ or left untreated) and analyzed by western blotting. Rab20 was associated with purified phagosomes during the first 30 min at relatively low levels (Fig. 1E). Strikingly, the association of Rab20 with phagosomes significantly increased after IFN-γ treatment. Thus, IFN-γ treatment increased the early recruitment of Rab20 to phagosomes (Fig. 1E,F). As expected, Rab5a was also associated with early phagosomes and dissociated at 60 min (Fig. 1E). Moreover, IFN-γ increased and extended the recruitment of Rab5a to phagosomes (Fig. 1E). Immunogold labeling on thawed cryosections of macrophages confirmed that IFN-γ enhanced the recruitment of Rab20 onto phagosomal membranes (Fig. 1G,H). Taken together, our data indicate that IFN-γ stimulates the recruitment of Rab20 to phagosomes at the very early steps after internalization.

### EGFP–Rab20 association with phagosomes delays phagosome maturation

To investigate the dynamics of Rab20 association in the context of phagosome maturation, we performed live-cell imaging in macrophages expressing fluorescently tagged wild-type Rab20 or the dominant-negative form of Rab20, Rab20T19N. A constitutively active mutant of Rab20 was not used in these studies because Rab20 has an unusual arginine in place of the glutamine at position 59 in the alpha-3 (switch II) region. Therefore, the GTPase activity of this mutant requires further characterization before its use in functional studies (data not shown).

**Fig. 2.**
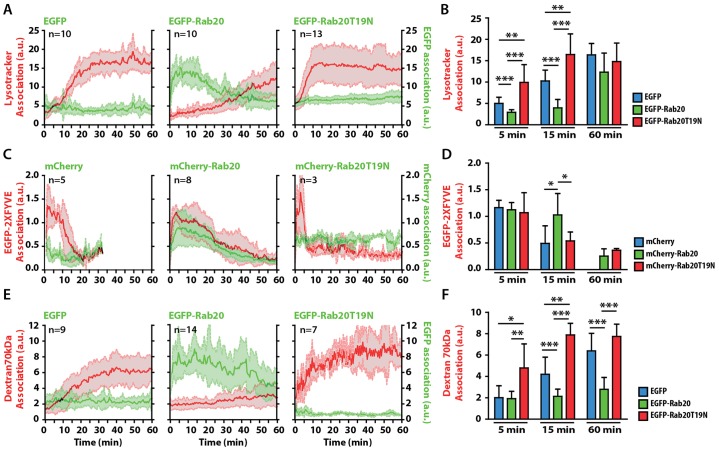
**The association of Rab20 with phagosomes delays phagosome maturation.** (A) The kinetics of LTR acquisition by phagosomes in macrophages expressing EGFP, EGFP–Rab20 or EGFP–Rab20T19N. RAW264.7 macrophages were transfected with EGFP, EGFP–Rab20 or EGFP–Rab20T19N and incubated with LTR for 30 min. Cells were subsequently incubated with 3-µm IgG-coated beads and analyzed by using live-cell imaging. (B) Data points from A at 5, 15 and 60 min were pooled. (C) The kinetics of the association of PI3P with phagosomes in macrophages expressing mCherry, mCherry–Rab20 or mCherry–Rab20T19N. RAW264.7 macrophages were co-transfected with EGFP–2xFYVE and mCherry, mCherry–Rab20 or mCherry–Rab20T19N, incubated with 3-µm IgG-coated beads and analyzed by using live-cell imaging. EGFP–2xFYVE and mCherry fluorescence were pseudo-colored to red and green, respectively. The intensity of both EGFP and mCherry fluorescence was normalized to the fluorescence intensity of the cytoplasm. (D) Data points from C at 5, 15 and 60 min were pooled. (E) The kinetics of the delivery of 70-kDa dextran to phagosomes in macrophages expressing EGFP, EGFP–Rab20 and EGFP–Rab20T19N. RAW264.7 macrophages were transfected with EGFP, EGFP–Rab20 or EGFP–Rab20T19N and preloaded with Texas-Red-conjugated 70-kDa dextran (50 µg/ml) for 2 h, followed by washing and chasing for 16 h. Afterwards, cells were incubated with 3-µm IgG-coated beads and analyzed by using live-cell imaging. (F) The data points from E at 5, 15 and 60 min were pooled. *n*, the number of phagosomes analyzed in at least three different experiments. All data show the mean±s.d. of the intensity at 5, 15 and 60 min post-internalization from three independent experiments. *P≤0.05, **P≤0.01, ***P≤0.001 (Student's two-tailed unpaired *t*-test).

First, cells were labeled with Lysotracker Red (LTR), and the association of the fluorescent signal with phagosomes was measured at the single phagosome level. In EGFP-expressing macrophages, phagosomes acquired LTR at ∼10 min and reached a plateau after 25 min (Fig. 2A,B; supplementary material Movie 1). In macrophages expressing EGFP–Rab20, phagosomes did not acquire LTR during the first 25 min, and this time-window correlated precisely with the period in which EGFP–Rab20 was present on phagosomes. After EGFP–Rab20 dissociated from the phagosomes, they started to acquire LTR and reached LTR levels that were comparable to those of the EGFP-expressing control after 60 min (Fig. 2A,B; supplementary material Movie 1). This delaying effect was specific for Rab20, because the overexpression of EGFP–Rab5a did not affect LTR acquisition (supplementary material Fig. S1E–G). By contrast, in macrophages expressing EGFP–Rab20T19N, phagosomes had already acquired LTR at 5 min, reaching a plateau at 10 min (Fig. 2A,B; supplementary material Movie 1). When another marker of maturation, phosphatidylinositol 3-phosphate (PI3P) (Bohdanowicz et al., 2010; Vieira et al., 2001) was tested, we found, as shown previously (Vieira et al., 2001), that PI3P resided in phagosomes of mCherry-expressing macrophages for the first 10 min (Fig. 2C,D). However, in macrophages expressing mCherry–Rab20, the association of PI3P with phagosomes was significantly prolonged, correlating with the presence of EGFP–Rab20 on phagosomes (Fig. 2C,D). By contrast, the association of PI3P with phagosomes in Rab20T19N-expressing macrophages lasted only ∼5 min (Fig. 2C,D). Additionally, the delivery into phagosomes of Texas-Red-conjugated 70-kDa dextran (Dex70kDa) that had been preloaded into late endocytic organelles started after 10 min in EGFP-expressing macrophages (Fig. 2E,F). However, in macrophages expressing EGFP–Rab20, the delivery of Dex70kDa into phagosomes was completely blocked during the first 60 min (Fig. 2E,F). By contrast, in Rab20T19N-expressing macrophages, the delivery of Dex70kDa proceeded significantly faster than in EGFP-expressing cells. Collectively, these data indicate that early phagosome progression is delayed by Rab20 overexpression. By contrast, the expression of the dominant-negative form of Rab20 accelerates early phagosome maturation.

### The presence of EGFP–Rab20 on phagosomes extends the time for which Rab5a associates with phagosomes

**Fig. 3.**
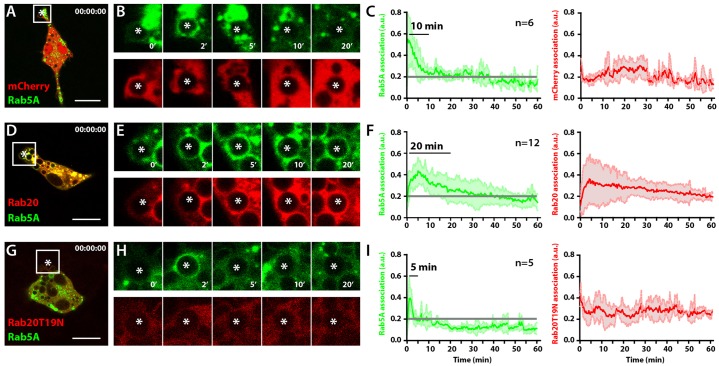
**The association of Rab20 with phagosomes extends the residence time of Rab5a.** (A) A snapshot of RAW264.7 macrophages coexpressing mCherry and EGFP–Rab5a at time 0, analyzed by live-cell imaging. (B) Time series showing the association of EGFP–Rab5a with the phagosome of interest (asterisk) in macrophages expressing mCherry and EGFP–Rab5a. (C) Quantitative analysis of the association of EGFP-Rab5a (left panel) and mCherry (right panel) with phagosomes. (D) A snapshot of RAW264.7 macrophages coexpressing mCherry–Rab20 and EGFP–Rab5a at time 0, analyzed by live-cell imaging. (E) Time series showing the association of EGFP–Rab5a with the phagosome of interest (asterisk) in macrophages expressing EGFP–Rab5a and mCherry–Rab20. (F) Quantitative analysis of the association of EGFP–Rab5a (left panel) and mCherry–Rab20 (right panel) with phagosomes. (G) A snapshot at time 0 of RAW264.7 macrophages coexpressing mCherry–Rab20T19N and EGFP–Rab5a at time 0, analyzed by live-cell imaging. (H) Time series showing the association of EGFP–Rab5a with the phagosome of interest (asterisk) in macrophages expressing EGFP–Rab5a and mCherry–Rab20T19N. (I) Quantitative analysis of the association of EGFP–Rab5a (left panel) and mCherry–Rab20T19N (right panel) with phagosomes. The intensity of both mCherry and EGFP signals were normalized to the fluorescence intensity of the cytoplasm. The data show the mean±s.d. from three (mCherry and mCherry–Rab20) or two (mCherry–Rab20T19N) independent experiments. *n*, the number of phagosomes analyzed in at least three different cells. Grey lines in panels C, F and I represent the background levels. Scale bars: 10 µm.

Because we observed that the association of Rab20 with phagosomes delayed phagosome maturation, we investigated the phagosomal dynamics of the early endocytic GTPase Rab5a. To do this, we used macrophages co-expressing EGFP–Rab5a with mCherry, mCherry–Rab20 or the dominant-negative mCherry–Rab20T19N. In cells expressing mCherry, EGFP–Rab5a was recruited early after phagosome formation and its levels decreased after 10 min, in agreement with previous studies (Lippuner et al., 2009; Roberts et al., 2006; Vieira et al., 2003) (Fig. 3A–C). Consistent with our previous observations, the expression of mCherry–Rab20 extended the time for which EGFP–Rab5a remained associated with phagosomes by a period of ∼20 min (Fig. 3D–F). By contrast, in macrophages expressing mCherry–Rab20T19N, EGFP–Rab5a was associated with phagosomes during the first 3–4 min after phagosome formation, followed by a rapid dissociation from phagosomes (Fig. 3G–I). Taken together, our data indicate that the association of Rab20 with phagosomes induced a prolonged association of the early phagocytic marker Rab5a with phagosomes.

### Rab20 knockdown accelerates the maturation of phagosomes and the dissociation of Rab5a

**Fig. 4.**
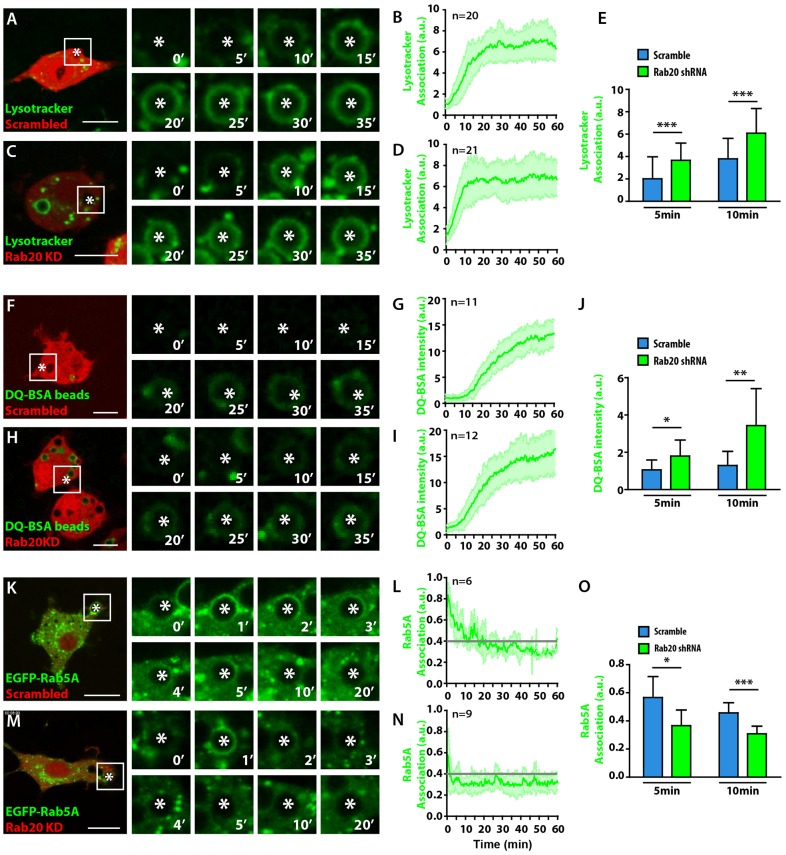
**Rab20 is required for a delay in phagosome maturation and for the recruitment of Rab5a.** (A) The kinetics of LTG acquisition by phagosomes in RAW264.7 macrophages expressing scrambled shRNA. Cells were incubated with LTG for 30 min and were subsequently incubated with 3-µm IgG-coated beads and analyzed by live-cell imaging. (B) Quantitative analysis of the intensity of LTG association with phagosomes post-internalization. (C) The kinetics of LTG acquisition by phagosomes in macrophages expressing Rab20 shRNA (Rab20 KD). (D) Quantitative analysis of the intensity of LTG association with phagosomes post-internalization. (E) Data points from B and D were pooled. (F) The kinetics of phagosomal degradation of DQ-BSA-coated beads in macrophages expressing scrambled shRNA. (G) Quantitative analysis of the intensity of DQ-BSA-coated beads in phagosomes. (H) The kinetics of the phagosomal degradation of DQ-BSA in macrophages expressing Rab20 shRNA. (I) Quantitative analysis of the intensity of DQ-BSA in phagosomes. (J) Data points from G and I were pooled. (K) The kinetics of the association of Rab5a with phagosomes in macrophages expressing scrambled shRNA. (L) Quantitative analysis of the intensity of the EGFP–Rab5a association with phagosomes post-internalization. (M) The kinetics of the association of Rab5a with phagosomes in macrophages expressing Rab20 shRNA. (N) Quantitative analysis of the intensity of EGFP–Rab5a association post-internalization. (O) Data points from L and N were pooled. White asterisks, the phagosome of interest. Scale bars: 10 µm. *n*, the number of phagosomes analyzed in at least three different cells. Grey lines in panels L and N represent the background levels. The data show the mean±s.d. from at least three independent experiments. *P≤0.05, **P≤0.01, ***P≤0.001 (Student's two-tailed unpaired *t*-test).

To provide independent support for the observations described above, we used the pSIREN-dsRed system to express small hairpin RNAs (shRNAs) in order to knock down Rab20 (Kasmapour et al., 2012). Of all the Rab20 shRNAs tested, shRNA1 and shRNA4 showed the best knockdown efficiency, and we used shRNA1 for experiments (supplementary material Fig. S2). RAW264.7 macrophages were transfected with vector expressing either scrambled shRNA or shRNA1, and phagosome maturation was analyzed by measuring Lysotracker Green DND-26 (LTG) acquisition as described above for LTR. Consistent with our dominant-negative approach, the acquisition of LTG proceeded significantly faster in macrophages expressing Rab20 shRNA compared with the scrambled shRNA (Fig. 4A–E; supplementary material Movie 2). Next, we evaluated the phagosomal proteolytic activity by measuring the green fluorescence intensity of self-quenched DQ Green BSA (DQ-BSA)-labeled beads (Yates et al., 2007). The phagosomal degradation of DQ-BSA proceeded significantly faster in macrophages expressing Rab20 shRNA compared with the scrambled shRNA (Fig. 4F–J). Finally, we analyzed the recruitment of EGFP–Rab5a in cells knocked down for Rab20. In agreement with the experiments performed in macrophages expressing EGFP–Rab20T19N, in cells expressing shRNA1, the early acquisition of EGFP–Rab5a by phagosomes was severely impaired compared with the cells expressing scrambled shRNA (Fig. 4K–O). Taken together, our data indicate that Rab20 is required for an early delay in both phagosome maturation and proteolytic activity, as well as for Rab5a recruitment into phagosomes.

### Role of IFN-γ and Rab20 in delaying phagosome maturation

**Fig. 5.**
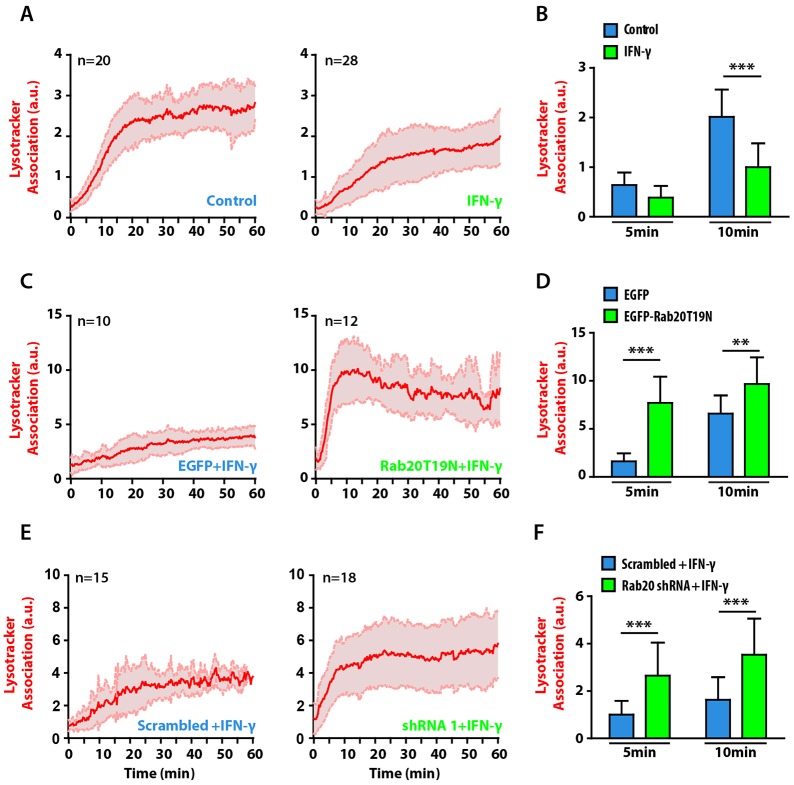
**Rab20 is required for the IFN-γ-induced delay in phagosome maturation.** (A) The kinetics of LTR acquisition by phagosomes in RAW264.7 macrophages that were untreated (left panel) or treated with 200 U/ml IFN-γ (right panel). The cells were incubated with LTR for 30 min and subsequently incubated with 3-µm IgG-coated beads and analyzed by using live-cell imaging. Two independent experiments were performed. (B) Data points from A at 5 and 10 min were pooled. (C) The kinetics of LTR acquisition by phagosomes in macrophages expressing EGFP (left panel) or EGFP–Rab20T19N (right panel) after IFN-γ treatment. (D) Data points from C at 5 and 10 min were pooled (E) The kinetics of LTG acquisition by phagosomes in macrophages expressing scrambled shRNA (left panel) or Rab20 shRNA (right panel). (F) Data points from E at 5 and 10 min were pooled. The data show the mean±s.d. from at least three independent experiments. *n*, number of phagosomes analyzed. **P≤0.01, ***P≤0.001 (Student's two-tailed unpaired *t*-test).

Little is known about the molecular players involved in the delay of early phagosome maturation that is induced by IFN-γ. Based on our results, we tested whether Rab20 could be the link between IFN-γ and the early maturation delay of phagosomes. As reported previously (Yates et al., 2007), in IFN-γ-treated macrophages, the majority of phagosomes displayed a significant delay in LTR acquisition relative to non-stimulated cells (Fig. 5A,B). Strikingly, phagosome maturation was not delayed by IFN-γ treatment of cells expressing the Rab20 dominant-negative mutant, relative to cells expressing only EGFP (Fig. 5C,D; supplementary material Movie 2). Accordingly, the effect of IFN-γ in delaying LTG acquisition in macrophages expressing scrambled shRNA was abolished in macrophages that were knocked down for Rab20 (Fig. 5E,F; supplementary material Movie 3). Taken together, our data indicate that the early delay in phagosome maturation induced by IFN-γ is dependent on Rab20.

### GTP–Rab20 interacts with Rabex-5

**Fig. 6.**
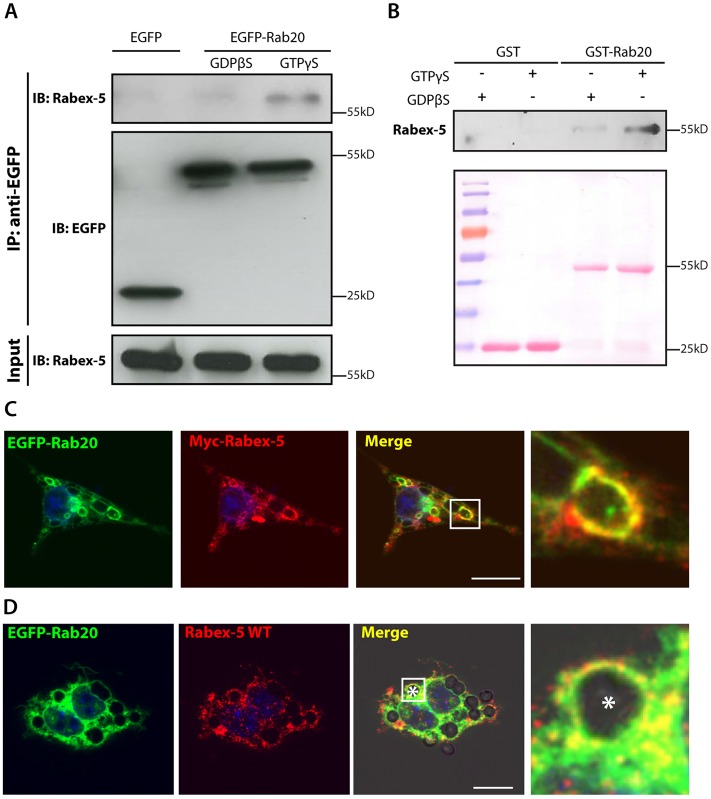
**Rabex-5 is an effector of Rab20.** (A) Co-immunoprecipitation of Rabex-5 from EGFP–Rab20-expressing macrophages. Macrophages stably expressing EGFP–Rab20 were lysed in buffer supplemented with either GTPγS or GDPβS and incubated with GFP–Trap® beads. Lysates were analyzed by SDS-PAGE, and EGFP and Rabex-5 were detected by western blotting. (B) The *in vitro* interaction of Rabex-5 and GTP–Rab20. GST or GST–Rab20 were immobilized on agarose beads, preloaded with either GTPγS or GDPβS and incubated with a lysate from RAW264.7 macrophages. After washing, the beads were collected, analyzed by SDS-PAGE and Rabex-5 was detected by western blotting. Ponceau S staining (lower panel) shows protein loading. (C) Colocalization of EGFP–Rab20 and wild-type (WT) Myc–Rabex-5 in macrophages. RAW264.7 macrophages were transfected with EGFP–Rab20 and wild-type Myc–Rabex-5 and subsequently fixed. (D) Colocalization of EGFP–Rab20 and wild-type Myc–Rabex-5 on phagosomes. RAW264.7 macrophages were transfected with EGFP–Rab20 and wild-type Myc–Rabex-5 and subsequently incubated with 3-µm IgG-coated beads. Nuclei were stained with Hoechst 33258 and are shown in blue. Magnified images show the regions of interest (indicated by white squares in the lower-magnification images). White asterisk, the phagosome. Scale bars: 10 µm.

We hypothesized that the effect of Rab20 overexpression in extending the time for which Rab5a remains associated with phagosomes might be due to the interaction of Rab20 with the exchange factor of Rab5a, Rabex-5. To test this hypothesis, we investigated whether Rabex-5 and Rab20 interact in macrophages expressing EGFP or EGFP–Rab20. Macrophages were lysed in the presence of either 1 mM GTPγS or 1 mM GDPβS, then immunoprecipitated using an anti-GFP antibody and immunoblotted for either GFP or Rabex-5 (Fig. 6A). Endogenous Rabex-5 was preferentially associated with EGFP–Rab20–GTPγS, indicating that Rabex-5 preferentially interacts with the GTP-bound form of Rab20 (Fig. 6A). Pull-down assays using RAW264.7 cell lysates confirmed that Rabex-5 specifically binds to GST–Rab20–GTPγS (Fig. 6B). Moreover, we found that in cells co-expressing Myc–Rabex-5 (Mattera and Bonifacino, 2008) and EGFP–Rab20, Rabex-5 partially colocalized with Rab20 in macrophages (Fig. 6C) and on early phagosomes (Fig. 6D). Taken together, these results indicate that the Rab5a GEF Rabex-5 also interacts specifically with the active form of Rab20.

### Rab20 recruits Rabex-5 into phagosomes in an IFN-γ-dependent manner

**Fig. 7.**
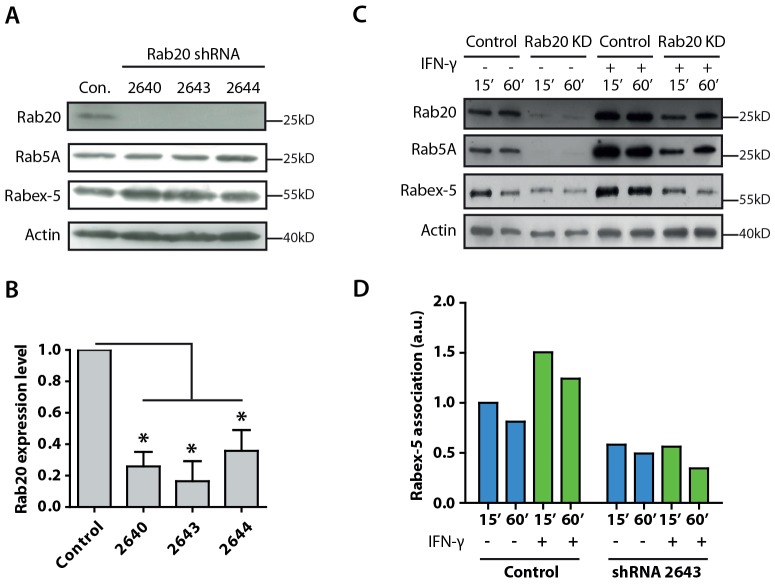
**Rab20 mediates the recruitment of Rabex-5 into phagosomes in IFN-γ-activated macrophages.** (A) Western blot analysis of Rab20, Rab5a and Rabex-5 levels in macrophages stably expressing Rab20 shRNAs (2640, 2643 and 2644). Actin was used as a loading control. Con, control. (B) Quantitative analysis of the expression level of Rab20 in macrophages stably expressing Rab20 shRNAs. The intensity was calculated relative to the actin loading control and normalized to the relative intensity in control samples. The data show mean±s.e.m. of three independent experiments. *P≤0.05 (Student's two-tailed *t*-test). (C) Western blot analysis of Rab20, Rab5a and Rabex-5 levels in isolated phagosomes from control and Rab20-knockdown (KD) macrophages with or without IFN-γ treatment. (D) Quantitative analysis of the association of Rabex-5 with phagosomes in control and Rab20-knockdown macrophages with or without IFN-γ treatment. The relative Rabex-5 intensity was compared with the actin loading control and normalized against the 15 min time-point.

Next, we investigated whether Rabex-5 is recruited to phagosomes through the Rab20 pathway in IFN-γ-stimulated macrophages. To analyze Rabex-5 recruitment, we generated stable Rab20-knockdown macrophages using a different strategy. RAW264.7 macrophages were transfected with pLKO.1 from which specific shRNAs targeting Rab20 were expressed, and stable knockdown cells were selected by using puromycin. The shRNA 2643 showed the best knockdown efficiency (Fig. 7A,B) and, hence, cells stably expressing shRNA2643 were used for the isolation of latex-bead phagosomes.

In resting macrophages, confirming our single-cell analysis in Rab20-knockdown macrophages (Fig. 4M,N), depletion of Rab20 impaired the recruitment of Rab5a into phagosomes (Fig. 7C,D). Rabex-5 was recruited to phagosomes, and its association with phagosomes was significantly decreased in Rab20-knockdown macrophages (Fig. 7C). This was not due to any significant reduction in total Rab5a or Rabex-5 levels in Rab20-knockdown cells, because the levels of both proteins remained unchanged (Fig. 7A). After IFN-γ stimulation, the recruitment of Rab5a to phagosomes was significantly impaired in Rab20-knockdown macrophages (Fig. 7C). Moreover, the association of Rabex-5 with phagosomes significantly increased after IFN-γ stimulation. This IFN-γ-stimulated association of Rabex-5 with phagosomes was impaired in macrophages that were silenced for Rab20 (Fig. 7C,D). Taken together, these results indicate that IFN-γ stimulates the association of Rab20 with phagosomes, leading to the recruitment of Rabex-5 that, in turn, activates Rab5a on phagosomes.

## DISCUSSION

Here, we have elucidated part of the molecular machinery by which IFN-γ modulates the early steps of phagosome maturation, and we have identified Rab20 as a key Rab protein in this pathway. First, IFN-γ increases the early recruitment of Rab20 to phagosomes, which has functional consequences. Previous studies have suggested that Rab20 might function in phagosome maturation (Egami and Araki, 2012; Gutierrez et al., 2008; Seto et al., 2011). Using three different approaches to monitor phagosome progression by live-cell imaging, we demonstrated that LTR acquisition, PI3P dissociation and cargo delivery are all transiently impaired when high levels of Rab20 are associated with phagosomes. The expression of Rab5a is stimulated by IFN-γ (Alvarez-Dominguez and Stahl, 1998); however, we observed that the effect of Rab20 is specific, because the overexpression of Rab5a, which, like Rab20, is associated with early phagosomes, does not modify phagosome maturation rates. By contrast, the expression of the dominant-negative mutant Rab20T19N and Rab20 knockdown accelerates phagosome maturation. In contrast to our results, it has been reported that LTR acquisition and cathepsin D recruitment to phagosomes is reduced in chemically fixed cells expressing the Rab20 dominant-negative mutant (Egami and Araki, 2012; Seto et al., 2011). These conflicting data might, in part, arise from differences between experimental systems. Monitoring phagosome maturation is difficult due to the asynchronous and dynamic nature of phagosomal interactions. Thus, live-cell imaging is crucial to visualize rapid and transient changes of individual phagosomes. Because Rab20 is associated with phagosomes at a very early stage, we focused on the events occurring during this phase in live macrophages. This was important because Rab20 and Rab20T19N expression had no effect at later time-points e.g. after 2 h of internalization, as observed here, and as reported previously (Egami and Araki, 2012).

Several lines of evidence support the idea that, in macrophages, IFN-γ induces an early delay in phagosome maturation. It has been observed that, in IFN-γ-activated macrophages, there is a delay in the progression of phagosomes to phagolysosomes (Tsang et al., 2000). The acidification rate, proteolytic activity (Yates et al., 2007) and the acquisition of LAMP-1, lysozyme-C and β-hexosaminidase (Jutras et al., 2008; Trost et al., 2009) by early phagosomes is reduced by IFN-γ. Here, we provide functional evidence indicating that the reported delay in maturation and proteolytic activity is, in part, due to increased levels of Rab20 in phagosomes. Rab20, in turn, transiently recruits Rabex-5, thereby increasing the levels of active Rab5a on phagosomal membranes. Compelling evidence also indicates that the early phagosomal delay has an important physiological function. In macrophages, relative slow phagosomal acidification and antigen degradation is crucial for efficient peptide antigen cross-presentation (Jutras et al., 2008; Trost et al., 2009). Even very subtle differences in acidification have a profound impact on proteolysis events occurring in phagosomes (Jiang et al., 2012). Our observations support the idea that small temporal changes mediated by Rab20 during phagosome maturation result in crucial changes in its function. However, the relevance of Rab20 function to antigen presentation or other phagosomal activities remains to be demonstrated. We postulate that IFN-γ has at least two effects on phagosomal functions; one is the relatively early delay in phagosome maturation and the second, operating later, is the enhancement of phagosomal maturation. In this context, Rab20 contributes to the early phagosomal functions of IFN-γ (supplementary material Fig. S3).

According to the data presented here, the Rab5a nucleotide exchange factor Rabex-5 is also an effector of Rab20. This is not the first time that a specific effector is identified as being shared by more than one Rab protein (Colucci et al., 2005; De Renzis et al., 2002; Yamamoto et al., 2010). Systems in which one particular Rab GTPase recruits an effector that sequentially modifies another Rab protein that is present in the same organelle have been reported in multiple trafficking pathways (Hutagalung and Novick, 2011; Pfeffer, 2013). The results of proteomic studies performed on isolated latex-bead phagosomes suggested that multiple Rab cascades coexist in this organelle (Gutierrez, 2013). Here, our data reveal one ‘phagosomal Rab cascade’ that involves the recruitment of Rab20, which in turn recruits the Rab5a GEF Rabex-5. This recruitment temporally increases the levels of Rab5a in phagosomes, extending the positive Rabex-5–Rab5a feedback, thereby transiently delaying phagosome maturation. Thus, we suggest that the Rab20-dependent recruitment of Rabex-5 to phagosomes temporally enhances the Rabex-5–Rab5a positive feedback (supplementary material Fig. S3). Therefore, the time needed to override the positive feedback and to undergo the transition from Rab5 to Rab7 is longer (Poteryaev et al., 2010), leading to a transient delay in phagosome maturation. Clearly, more studies are needed to investigate how Rab5a activation is shut down and to determine the identity of the Rab proteins that mediate IFN-γ function at later stages.

Importantly, we demonstrate that this sequential recruitment is regulated by IFN-γ, a key cytokine for the activation of macrophages. Thus, our study contributes to a better understanding of the link between the immune activation of macrophages and the control of phagosome function. Our results suggest that similar mechanisms of cytokine control might exist for currently unidentified phagosome-associated Rab cascades. Such unidentified Rab cascades might operate during phagosome maturation to modulate the multiple immune functions of this organelle.

## MATERIALS AND METHODS

### Cells and reagents

RAW264.7 macrophages were obtained from the American Type Culture Collection (ATCC, catalog number TIB-71) and maintained in complete Dulbecco's Modified Eagles Medium (D-MEM, 4.5 g/l glucose) with 10% (v/v) heat-inactivated fetal calf serum (FCS, PAA, Pasching, Austria) and 2 mM l-glutamine (PAA). Bone marrow macrophages (BMMs) were isolated and maintained as described previously (Kasmapour et al., 2012). Cells were incubated at 37°C under 5% CO_2_ in a humidified incubator. For IFN-γ stimulation, RAW264.7 macrophages and BMMs were stimulated with 200 U/ml and 400 U/ml IFN-γ (Peprotech, Rocky Hill, NJ), respectively for 16–20 h.

### Antibodies

The following primary antibodies were used: rabbit anti-Rab20 (11616-1-AP, Proteintech, Chicago, IL); rabbit anti-Rab20 (GTX119559, Genetex, Irvine, CA); rabbit anti-Rab5a (C8B1, Cell Signaling Technology, Beverly, MA); rabbit anti-Rabex-5 (Sigma-Aldrich, Germany); mouse anti-β-actin (mAb8226, Abcam, Cambridge, UK); mouse anti-GFP (JL-8, Clontech, Mountain View, CA) and rabbit anti-c-Myc (A-14, Santa Cruz Biotechnology, Dallas, TX). Secondary antibodies conjugated to Alexa Fluor 488, 546 or 633 for indirect immunofluorescence studies were purchased from Molecular Probes (Invitrogen, Carlsbad, CA). Secondary antibodies conjugated to Cy3 or horseradish peroxidase (HRP) were obtained from Jackson ImmunoResearch Laboratories (West Grove, PA).

### Plasmids

The mouse Rab20 gene was amplified by standard PCR from the cDNA clone with specific primers and was cloned into the pEGFP-C1 vector (Clontech). The pEGFP-C1-Rab20T19N plasmid was generated by site-directed mutagenesis using the QuikChange II XL mutagenesis kit (Agilent Genomics, Santa Clara, CA). mCherry-Rab20 and mCherry-Rab20T19N plasmids were both subcloned from the pEGFP-C1-Rab20 and Rab20T19N plasmids, respectively, into the *EcoR*I and *BamH*I sites. All DNA sequences were confirmed by full sequencing. The LifeAct-RFP plasmid was obtained from Ibidi (Verona, WI). The EGFP-Rab5a plasmid was kindly provided by Philip D. Stahl (Washington University, St Louis, MO). The EGFP-2xFYVE plasmid was kindly provided by Mauricio Terebiznik (University of Toronto, Canada). The Myc-Rabex-5 plasmid was kindly provided by Rafael Mattera and Juan Bonifacino (NIH, Bethesda, MD). RNAi-Ready pSIREN-RetroQ-DsRed-Express vector was obtained from Clontech.

### Macrophage transfection

Cells were transfected by using Lipofectamine 2000 (Invitrogen). For transfection, 0.5×10^5^cells/well were seeded onto a 24-well plate 1 day before transfection. On the day of transfection, the cells were washed twice with pre-warmed Ca^2+^-Mg^2+^-free Dulbecco's phosphate buffered saline (PBS, PAA) and incubated with Opti-MEM reduced-serum medium (Invitrogen). 0.5 µg of plasmid DNA and 1 µl of Lipofectamine diluted in Opti-MEM were used for each well. After 6 h, the Opti-MEM medium was exchanged for complete D-MEM. For JetPEI-Macrophage transfection (Polyplus-transfection, France), 1×10^5^ cells were seeded onto a 35 mm^2^ glass-bottomed dish (MatTek, Ashland, MA) or a CELLview® glass-bottomed dish (Greiner Bio-One, Frickenhausen, Germany). 1 µg of plasmid DNA and 3 µl of JetPEI-Macrophage were used for each transfection. For both methods, cells were transfected 16 h before experiments.

### Bead preparation and phagosome isolation

Latex beads (1 µm or 3 µm in diameter, 2.5% solution, Polysciences, Warrington, PA) or 3-µm polystyrene beads (Krisker Biotech, Steinfurt, Germany) were coupled to 50 µg/ml mouse IgG (Rockland, Gilbertsville, PA) or 50 µg/ml DQ Green BSA (Life Technologies, Grand Island, NY) in 10% (v/v) 0.5 M 2-(N-morpholino)ethanesulfonic acid (MES) buffer pH 6.7 with 1-ethyl-3-(3-dimethylaminopropyl)carbodiimide (EDAC) crosslinker (Sigma-Aldrich). The coupling was performed at room temperature for 2 h on a rotating wheel. The reaction was stopped by using 1% Triton X-100 in 10 mM Tris-HCl buffer pH 9.4. The beads were washed with PBS and stored as a 1% suspension in PBS at 4°C.

### Latex-bead phagosome isolation

Phagosomes were isolated as described previously (Wähe et al., 2010). Briefly, RAW264.7 macrophages were grown in complete D-MEM supplemented with IFN-γ (200 U/ml) for 16 h. Then the macrophages were pulsed with 1 µm IgG-coated latex beads for 45 min and were isolated at 15 min, 30 min and 60 min of chasing. Phagosomes were collected, incubated in SDS sample buffer at 96°C for 10 min and stored at −20°C for further analysis by western blotting.

### Indirect immunofluorescence

Cells were fixed with 4% (v/v) paraformaldehyde (PFA, Electron Microscopy Sciences, Hatfield, PA) in PBS pH 7.4 for 10 min at room temperature, followed by incubation with pre-cooled methanol (J.T.Baker, Center Valley, PA) for 2 min at −20°C. After washing twice with PBS, cells were incubated with 50 mM glycin in PBS pH 7.4 for 10 min and afterwards with 1% bovine serum albumin (BSA, Sigma-Aldrich) in PBS for 10 min (Kasmapour et al., 2012). The primary and secondary antibodies were diluted in PBS at the indicated dilutions and were added to cells with incubation for 1 h at room temperature. 1 µg/ml Hoechst 33258 (Invitrogen) was used for nuclear staining. Cells were mounted with DAKO mounting medium (Dako Cytomation, Carpinteria, CA).

### Cryosectioning and immunolabeling

RAW264.7 macrophages were incubated with 1-µm latex beads coupled to mouse IgG for 1 h. The cells were fixed with 4% PFA in 200 mM HEPES pH 7.4 overnight. The cell pellet was embedded in 12% bovine gelatin (Sigma), cut into small cubes and infiltrated with 2.3 M sucrose overnight. Samples were vitrified in liquid nitrogen and 100-nm Tokuyasu sections were cut by using a Leica EM UC7 Cryo-ultramicrotome (Leica Microsystems, Vienna, Austria). Sections were transferred onto formvar- and carbon-coated transmission electron microscopy (TEM) grids. A solution of 1% gelatin from cold-water fish skin (Sigma-Aldrich) in PBS was used for the blocking of non-specific binding and the dilution of antibody and Protein-A–gold (PAG) (University Medical Centre, Utrecht, The Netherlands). Rabbit antibody against Rab20 (Genetex) was used at a 1∶10 dilution for 30 min. 10-nm PAG was used at 1∶50 for 30 min. Sections were embedded in 2% methyl cellulose with 0.2% uranyl acetate (Electron Microscopy Sciences) and analyzed by using a TEM microscope (Philips CM100, Philips, Amsterdam, The Netherlands). The images were recorded digitally by using a Quemsa TEM CCD camera (Olympus Soft Imaging Solutions, Münster, Germany) and iTEM software (Olympus Soft Imaging Solutions). To estimate the ratio of Rab20-labeled latex beads, images of every latex bead in a chosen area of the section (in one mesh window of the grid) were sampled systematically. In total, 70 beads were analyzed per sample.

### Rab20 knockdown with the pSIREN system

Using the Clontech online shRNA design tool, four oligos were designed against *Mus musculus Rab20*: 1, 5′-CTGACAGAAACAGCCAACA-3′; 2, 5′-CCAACAATGACTGCCTGTT-3′; 3, 5′-CACCCAAACAGACTAGATC-3′ and 4, 5′-CCGCTATCATCCTTACATA-3′. The oligos were synthesized by Eurofins MWG Operon (Munich, Germany) and cloned into the RNAi-Ready pSIREN-DsRed-Express vector according to the recommended protocol, followed by sequence verification. Macrophages were transfected with plasmids expressing the shRNAs mentioned above, or scrambled shRNA as control, by using JetPEI-Macrophage. After 48 h of transfection, macrophages were used for live-cell imaging. For western blotting, transfected macrophages from 25-cm^2^ flasks were sorted for DsRed by using a BD Aria II sorter (BD Biosciences, San Jose, CA). Sorted cells were lysed in SDS sample buffer (50 mM Tris-HCl pH 6.8, 2% SDS, 10% glycerol, 1% β-mercaptoethanol, 12.5 mM EDTA and 0.02% bromophenol blue) and blotted with anti-Rab20 antibody (Proteintech).

### Rab20 knockdown with the MISSION® shRNA system

The MISSION® shRNA system (Sigma-Aldrich) was used to obtain stable Rab20-knockdown cell lines. Five Rab20 shRNA oligos were tested, including shRNA2640 (5′-CTGACAGAAACAGCCAACAA-3′), shRNA 2641 (5′-CTATCATCCTTACATACGAT-3′), shRNA 2642 (5′-CTCCTCTTTGAAACCTTGTT-3′), shRNA 2643 (5′-CCTTTACAAGAAGATCCTGA-3′) and shRNA 2644 (5′-AAGATCCTGAAGTACAAGAT-3′). The control shRNA plasmid was pLKO.1-puro empty vector. Macrophages were transfected with the corresponding plasmids by using JetPEI Macrophage as described above. After 16 h of transfection, puromycin (3 µg/ml, MP Biomedicals, Santa Ana, CA) was added as the selective agent and the medium was changed every 2 days with the addition of fresh antibiotic. After 1–2 weeks the colonies were selected and expanded. Rab20 knockdown in the cells from the selected colonies was checked by western blotting.

### SDS-PAGE and western blotting

Cells were lysed in lysis buffer (20 mM Tris-HCl pH 7.5, 150 mM NaCl, 0.5 mM EDTA and 0.5% NP-40) supplemented with complete protease inhibitor cocktail (Roche, Basel, Switzerland) on ice for 10 min. After centrifugation at 16,000 ***g*** for 10 min, the supernatants were heated with SDS sample buffer at 96°C for 10 min. Proteins were separated on a 10% SDS gel and transferred onto nitrocellulose membranes. The membranes were incubated with the corresponding dilutions of primary and secondary antibodies in 5% skimmed milk (Carl Roth, Karlsruhe, Germany) diluted in PBS 0.1% Tween-20 for 1 h at room temperature. For western blotting of Rab20, the membranes were incubated with anti-Rab20 antibodies [Proteintech (in Fig. 7A,C; supplementary material Fig. S2) or GeneTex (in Fig. 1E)] in 5% skimmed milk in PBS containing 0.1% Tween-20 overnight at 4°C. The membranes were developed by using the ECL Detection Kit (GE Life Sciences, Pittsburg, PA), scanned and evaluated using ImageJ software. The antibody against β-actin was used as the loading control.

### Phagosome maturation analysis by live-cell imaging

For live-cell imaging, 1×10^5^ macrophages were seeded onto a 35-mm glass-bottomed dish (MatTek) or a CELLview® glass-bottomed dish (Greiner Bio-One). Transfections were performed as described above. The cells were washed with PBS and replaced with imaging medium (complete D-MEM without Phenol Red, PAA). 3-µm IgG-coated polystyrene beads were added shortly before imaging. Time-lapse images were acquired by using a Leica SP5 AOBS (Leica Microsystems) equipped with an environmental control chamber (EMBLEM, Germany). Single focal planes were monitored over time (*xyt* scanning mode) using a 63×/1.4 HCX-PLAPO oil objective, argon laser (488 nm), DPSS laser (561 nm) and HeNe laser (633 nm). Scanning frequencies of 200–400 Hz and a line averaging of 2–3 at a rate of 20 s per frame using photomultiplier (PMT) and/or hybrid detector system (HyD) were applied, and videos were recorded at a resolution of 1024 pixels ×1024 pixels. To determine the association of LTR or LTG with phagosomes, the cells were first transfected with the indicated plasmids by using JetPEI-Macrophage as indicated. At a defined time after transfection, 50 nM LTR or LTG was diluted in imaging medium and added to the cells 30 min before adding 3-µm IgG-coated polystyrene beads as indicated above. To determine the association of PI3P with phagosomes, cells were first co-transfected with EGFP-2xFYVE and mCherry, mCherry–Rab20 or mCherry–Rab20T19N by using JetPEI-Macrophage. After 16 h of transfection, IgG-coated beads were added before imaging. To determine the association of Rab5a with phagosomes, cells were co-transfected with EGFP–Rab5a and mCherry, mCherry–Rab20 or mCherry–Rab20T19N by using JetPEI-Macrophage. After 16 h of transfection, IgG-coated beads were added before imaging. To analyze 70-KDa dextran delivery, the cells were transfected with EGFP, EGFP–Rab20 or EGFP–Rab20T19N by using JetPEI-Macrophage. After 6 h of transfection, the cells were washed with PBS, and complete D-MEM containing 50 µg/ml Texas-Red-conjugated 70-KDa dextran was added for 2 h followed by 16 h of chase. IgG-coated beads were added and live-cell imaging was performed.

### Image analysis

The time-lapse images were exported to uncompressed AVI-formatted movies as single channels with the LAS AF software (Leica microsystems). The RGB-color movies were loaded into ImageJ (v1.43u) and transformed into 8-bit color movies (Image→ Color→ Split channels). The 8-bit color movies were then ready for further quantification. To quantify the fluorescence association with the phagosomes, the corresponding fluorescent-channel movies were loaded into ImageJ. A circle enclosing the phagosome in the brightfield image was drawn by using the ‘Elliptical selection’ tool. In ‘Set Measurements’, only ‘Area’ and ‘Integrated Density’ were selected. Subsequently, the integrated intensity inside the circle was measured image by image (Analyze→ Measure). The position and the size of the circle were adjusted manually with the movement of the phagosome in the different frames. In experiments on Lysotracker acquisition and 70-kDa dextran delivery, the absolute integrated intensity was plotted by using GraphPad Prism (GraphPad software, La Jolla, CA). In experiments on PI3P association and Rab5a association, the expression level of EGFP–2xFYVE and EGFP–Rab5a was different in the different biological replicates. To take this effect into account, the fluorescence intensity of EGFP–2xFYVE and EGFP–Rab5a was normalized to the intensity of a region of the same size in the cytoplasm. The fluorescence intensity of mCherry, mCherry–Rab20 or mCherry–Rab20T19N was also normalized to the intensity of a region of the same size in the cytoplasm as shown in supplementary material Fig. S4. To analyze endogenous Rab20 association with phagosomes in fixed cells, a similar methodology was used; a circle enclosing the phagosome in the brightfield image was drawn, and the integrated density of the circular region of interest (ROI) redirected to the Rab20 channel was measured as shown in supplementary material Fig. S4. The normalized data was plotted with GraphPad Prism v5.04.

### Co-immunoprecipitation

RAW264.7 macrophages stably transfected with EGFP and EGFP–Rab20 were grown in T-75 flasks. When the cells reached 90% confluency, they were scraped, collected and lysed with 200 µl of lysis buffer [20 mM Tris-HCl pH 7.5, 150 mM NaCl, 0.5% NP-40, 80 mM β-glycerol phosphate disodium salt pentahydrate (Sigma-Aldrich), 50 mM NaF (Carl-Roth), 5 mM EDTA and 5 mM MgCl_2_, with the addition of complete protease inhibitor cocktail] for 30 min on ice. For cells stably transfected with EGFP–Rab20, lysis was performed with 200 µl of lysis buffer supplemented with 1 mM GTPγS (EMD Chemicals, Rockland, MA) or GDPβS (Merck, Darmstadt, Germany) for 30 min on ice, followed by the addition of 5 mM MgCl_2_ for another 30 min on ice. All cell lysates were centrifuged at 16,000 ***g*** for 10 min at 4°C. 25 µl of GFP–Trap® beads were added into each supernatant and mixed on a rotator wheel at 4°C for 2 h. The beads were subsequently washed three times with washing buffer (20 mM Tris-HCl pH 7.5 and 150 mM NaCl), incubated in SDS sample buffer at 96°C for 10 min and then analyzed by western blotting.

### GST pulldown

The glutathione–agarose beads (GST Buster QF Glutathione, Amocol, Teltow, Germany) were equilibrated with binding buffer (25 mM Tris-HCl pH 7.5, 150 mM NaCl, 5 mM DTT, 10% glycerol and 0.1% Triton X-100). 100 µg of GST or GST–Rab20 (in 20 mM Tris-HCl pH 7.5) were added to 50 µl of equilibrated beads and incubated for 30 min at room temperature with rotation. The beads were centrifuged at 700 ***g*** for 1 min to remove the unbound proteins. For GTPγS or GDPβS loading, the beads were incubated with 400 µl of nucleotide exchange buffer (20 mM Tris-HCl pH 7.5, 150 mM NaCl, 1 mM DTT, 5 mM EDTA, 10 mM MgCl_2_ and 2 mM GTPγS or GDPβS) for another 30 min at room temperature with rotation. For each T-175 flask, cells were lysed with 400 µl of lysis buffer (20 mM Tris-HCl pH 7.5, 150 mM NaCl, 0.5% NP-40, 80 mM β-glycerol phosphate disodium salt pentahydrate, 50 mM NaF, 5 mM EDTA, 5 mM MgCl_2_, 2 mM GTPγS or GDPβS and complete protease inhibitor cocktail) on ice for 30 min. Cell debris was removed by centrifuging at 16,000 ***g*** at 4°C for 10 min. Subsequently, the beads immobilized with GST (GTPγS), GST (GDPβS), GST–Rab20 (GTPγS) or GST–Rab20 (GDPβS) were incubated with the resulting supernatants for 2 h at 4°C with rotation. The beads were washed with washing buffer (20 mM Tris-HCl pH 7.5 and 150 mM NaCl) three times, incubated in SDS sample buffer at 96°C for 10 min and then analyzed by western blotting. The membranes were stained with Ponceaus S staining solution [0.1% (w/v) Ponceau S (Carl Roth) in 5% (v/v) acetic acid] for the loading control.

### Statistical analysis

Statistical analysis was performed in GraphPad Prism v5.04. *P*-values were calculated by using Student's *t*-test or the Mann-Whitney test. The confidence interval used is 95%.

## Supplementary Material

Supplementary Material
