# Supplementary material for: Identification of an immune-regulated phagosomal Rab cascade in macrophages
Source: J Cell Sci. 2014 May 1;127(9):2071–82. doi: 10.1242/jcs.144923 (PMC4004979; doi:10.1242/jcs.144923)
Supplement: Supplementary Material [file supp_127_9_2071__index.html]

Identification of an immune-regulated phagosomal Rab cascade in macrophages — Supplementary Material 

# Identification of an immune-regulated phagosomal Rab cascade in macrophages

## JCS144923 Supplementary Material

**Files in this Data Supplement:**

- **Supplementary Material**
