# Supplementary material for: Identification of an immune-regulated phagosomal Rab cascade in macrophages
Source: J Cell Sci. 2014 May 1;127(9):2071–82. doi: 10.1242/jcs.144923 (PMC4004979; doi:10.1242/jcs.144923)
Supplement: Supplementary Material [file supp_127.9.2071_JCS144923.pdf]

Supplementary information

Identification of an immune regulated phagosomal Rab cascade in macrophages

Supplementary legends

Figure S1- Rab20 dynamics during phagocytosis.

A. RAW264.7 macrophages were co-transfected with LifeAct-RFP (to visualize F-Actin *in vivo*) and EGFP-Rab20 and subsequently incubated with 3 µm IgG-coated beads. A snapshot from a representative time-lapse at time 0 is shown. On the right panel, the time series of images show F-Actin and EGFP-Rab20 dynamics during phagocytosis of a bead (indicated by the asterisk). Scale bar: 10 µm. Rab20 was recruited to phagosomes shortly after phagocytic cup closure. Actin was assembled at the point of bead attachment without Rab20 association during formation of the phagocytic cup (0 min). Rab20 rapidly associated with phagosomes after actin de-polymerization (1-3 min). Once internalized, F-actin quickly dissociated from phagosomes (5 min). **B.** Quantitative analysis of the fluorescence intensity of F-actin and Rab20 associated with the phagosome. **C.** Dynamic association of EGFP-Rab20 with phagosomes. RAW264.7 macrophages were transfected with EGFP-Rab20, subsequently incubated with 3 µm Cy5 conjugated IgG-coated beads (Red) and analyzed by live cell imaging. A representative snapshot of a time-lapse at time 0 is shown. Right panel: time series insets show EGFP-Rab20 association with phagosome (enclosed by the white square). Scale bar: 10 µm. **D.** Quantitative analysis of EGFP-Rab20 association with phagosomes during the first 35 min after internalization. The intensity of green fluorescence (EGFP-Rab20) associated with phagosomes in at least 6 phagosomes from a representative experiments was analyzed. Data show mean ± SD of EGFP-Rab20 fluorescence intensity associated with phagosomes. **E.** RAW264.7 macrophages were transfected with EGFP-Rab5a for 16 h and LTR (50 nM) was added 30 min prior to live cell imaging. An image from a representative movie is shown here. Scale bar: 10 µm. **F.** Kinetics of EGFP-Rab5a (Green) and LTR (Red) associated with phagosomes during the first 60 min of internalization. Association of LTR and EGFP-Rab5a with phagosomes was analyzed frame by frame. Data show mean ± SD of the intensity of LTR and EGFP-Rab5A associated with phagosomes. Five phagosomes of two cells in two independent experiments were quantified. **G.** Time series of the area indicated by white square in C showing a phagosome (indicated by the asterisk) acquiring LTR (Red) and EGFP-Rab5A (Green). Scale bar: 3 µm.

Figure S2- Rab20 knockdown with pSIREN shRNAs

RAW264.7 macrophages were transfected with pSIREN plasmid expressing scrambed shRNA, shRNA 1 or shRNA 4 for 30 h, and subsequently cells expressing these shRNAs were sorted by Fluorescence-activated cell sorting (FACS). The sorted cells were incubated in sample buffer at 96°C for 10 min. The lysates were blotted against Rab20 and actin as the loading control. The relative intensity of Rab20 was calculated referring to actin. The relative intensity of Rab20 in the lysate of scrambled shRNA was set as 100%. Data show mean ± SEM from at least three independent experiments. *P* values were calculated using student' two-tailed t-test. (*) $p \leq 0.05$, (**) $p \leq 0.01$.

Figure S3- Proposed model of Rab20 function in phagosome maturation

After IFN-γ stimulation, the expression of Rab20 increases and Rab20 is targeted to the phagosome. Rab20 induces the recruitment of the Rab5 GEF, Rabex-5 to phagosomes. Rabex-5 association to phagosomes then transiently increases the levels of active Rab5A on phagosomes. The extended association of Rab5A to phagosomes results in a temporal delay in maturation of phagosomes. Additionally, IFN-γ stimulation enhances maturation of phagosomes at later stages but the mechanism is not known.

Figure S4- Procedures of quantifying fluorescence intensity associated with bead-phagosomes in fixed and live cells. First, TIFF files (in case of fixed cells) or AVI files (in case of live cell imaging) were loaded into ImageJ and the different channels (green, red and bright field) split. All the images acquired under the different replicates and conditions had same magnification, so the scales were not taken into account. **1-3.** The scale is removed using the command “Analyze → Set Scale → Click to Remove Scale”. In step 3, “Global” is also selected. **4-5.** The parameters for measurement are adjusted using the command “Analyze → Set Measurements”. ”Area” and “Integrated Density” are selected as indicated in the upper rectangle of step 5. The measurement is redirected to the channel that needs to be quantified. Here as an example, it is redirected to the red channel as indicated in the lower rectangle of step 5. **6.** Circular ROIs (3 µm diameter ± 2 pixels) is draw around the selected bead phagosome in the bright field channel using “Elliptical selection tool”. Only beads in the right focus were quantified. **7.** The fluorescence intensity in the circular ROIs of the redirected channel is measured by using the command “Analyze → Measure”. For live cell imaging, the position and the size of the ROI in the bright field channel are adjusted manually with the movement of the phagosome in different frames. In experiments of

Lysotracker acquisition, Dextran70kDa delivery and DQ Green BSA degradation, the absolute integrated intensity was plotted with GraphPad Prism. **8.** In experiments of PI3P association and Rab5A association, the expression levels of EGFP-2XFYVE and EGFP-Rab5A were different between the biological replicates. To take into account this effect, a circular ROI with the exact same size in the cytoplasm in the channel of EGFP-2XFYVE or EGFP-Rab5A was drawn and the integrated density of the ROI measured. Next, the fluorescence intensity of the phagosomes was normalized with the corresponding intensity in the cytoplasm. The normalized data was plotted using GraphPad Prism.

Movie S1- LTR acquisition by phagosomes in macrophages expressing EGFP, EGFP-Rab20 or EGFP-Rab20T19N.

RAW264.7 macrophages were transfected with EGFP, EGFP-Rab20 or EGFP-Rab20T19N for 16 h and LTR (50 nM) was added 30 min prior to live cell imaging. Confocal images were recorded at one frame per every 20 seconds. Then color channels of original time-lapse movies were split and cropped to show one phagosome only in red channel for each movie. Movies are visualized in the 16-colors lookup table from ImageJ software. This video is shown at 15 frames per second and corresponds to Fig. 2A and B.

Movie S2- LTG acquisition by phagosomes in macrophages expressing scramble vs. Rab20 shRNA

RAW264.7 macrophages were transfected with pSIREN plasmid harboring scrambled shRNA or shRNA 1 for 30 h and LTG (50 nM) was added 30 min prior to live cell imaging. Confocal images were recorded at one frame per every 20 seconds and processed as indicated above. This video is shown at 15 frames per second and corresponds to Fig.4A-E.

Movie S3- LTG acquisition by phagosomes in macrophages expressing scrambled vs. Rab20 shRNA after IFN-γ treatment

RAW264.7 macrophages were transfected with pSIREN plasmid harboring scrambled shRNA or shRNA 1 for 24 h and subsequently treated with 200 U/ml IFN-γ for 16 h. LTG (50 nM) was added 30 min prior to live cell imaging. Confocal images were recorded at one frame per every 20 seconds and processed as indicated above. This video is shown at 15 frames per second and corresponds to Fig. 5E and F.

Figure S1

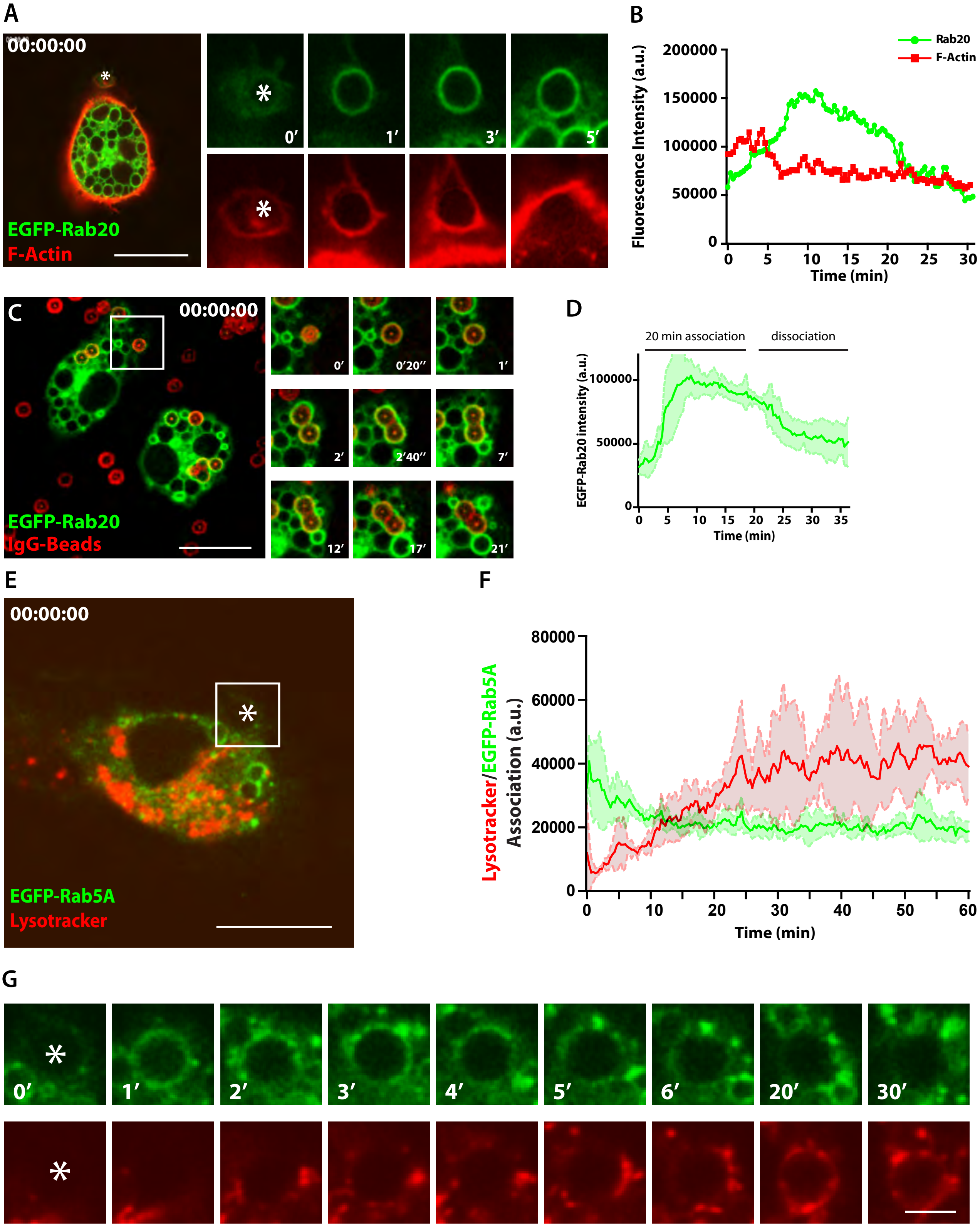
A
00:00:00
EGFP-Rab20
F-Actin
0′
1′
3′
5′
B
Rab20
F-Actin
Fluorescence Intensity (a.u.)
200000
150000
100000
50000
0
0 5 10 15 20 25 30
Time (min)
C
00:00:00
EGFP-Rab20
IgG-Beads
0′
0′20′′
1′
2′
2′40′′
7′
12′
17′
21′
D
20 min association
dissociation
EGFP-Rab20 intensity (a.u.)
100000
50000
0
0 5 10 15 20 25 30 35
Time (min)
E
00:00:00
EGFP-Rab5A
Lysotracker
F
Lysotracker/EGFP-Rab5A
Association (a.u.)
80000
60000
40000
20000
0
0 10 20 30 40 50 60
Time (min)
G
0′
1′
2′
3′
4′
5′
6′
20′
30′

Figure S2

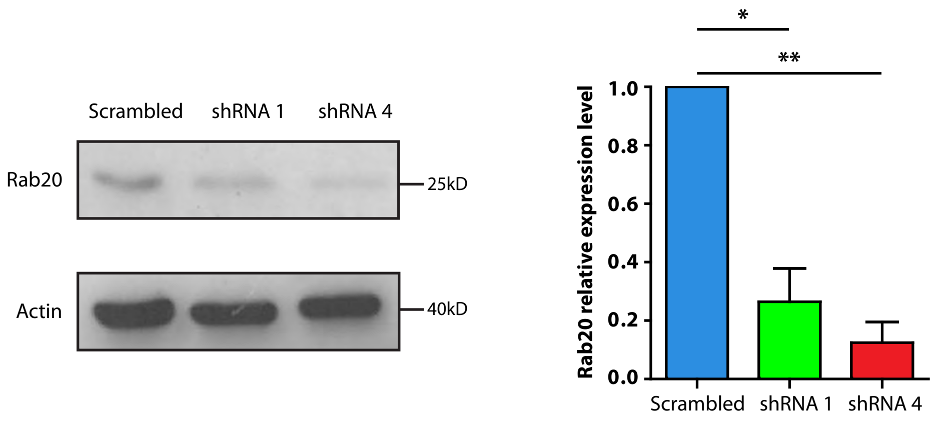

Scrambled
shRNA 1
shRNA 4
Rab20
Actin
25kD
40kD
*
**
Rab20 relative expression level
1.0
0.8
0.6
0.4
0.2
0.0
Scrambled
shRNA 1
shRNA 4

Figure S3

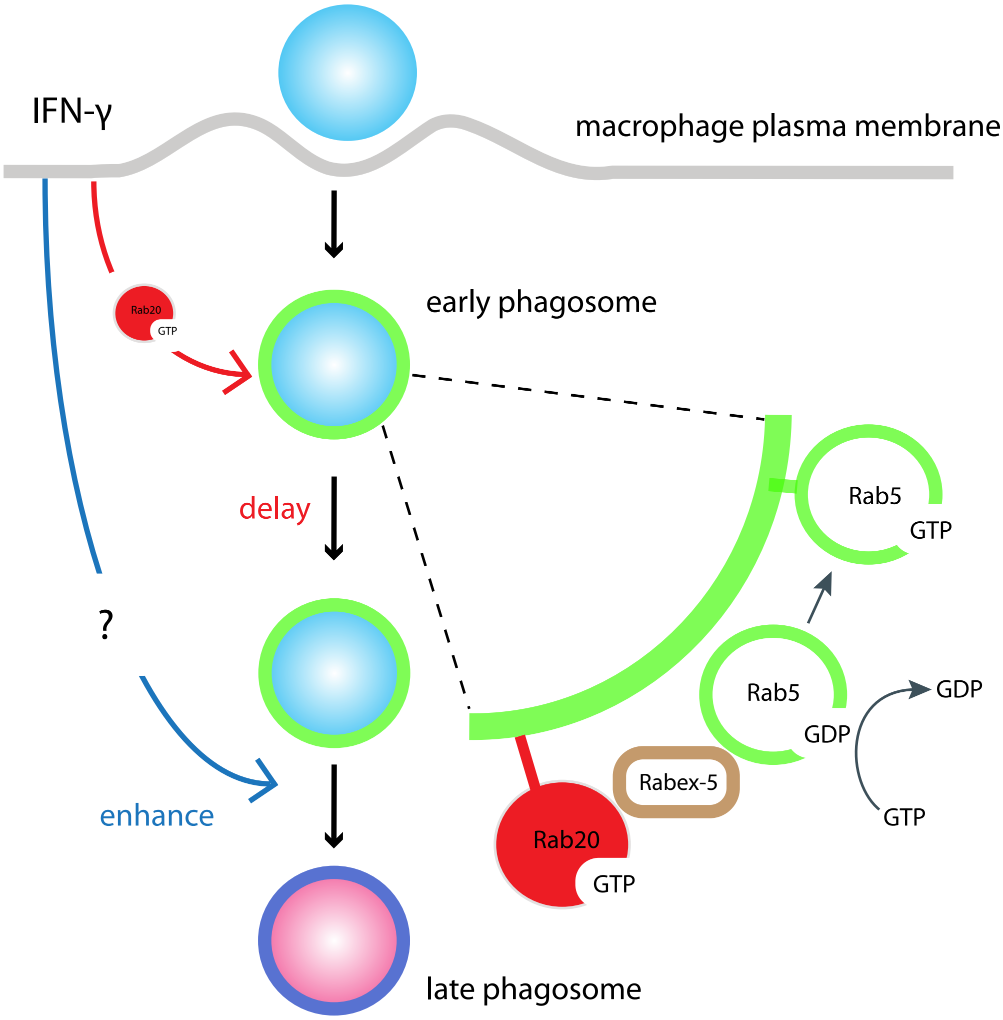
IFN-γ
macrophage plasma membrane
Rab20
GTP
early phagosome
delay
?
enhance
late phagosome
Rab5
GTP
Rab5
GDP
GDP
GTP
Rabex-5
Rab20
GTP

Figure S4

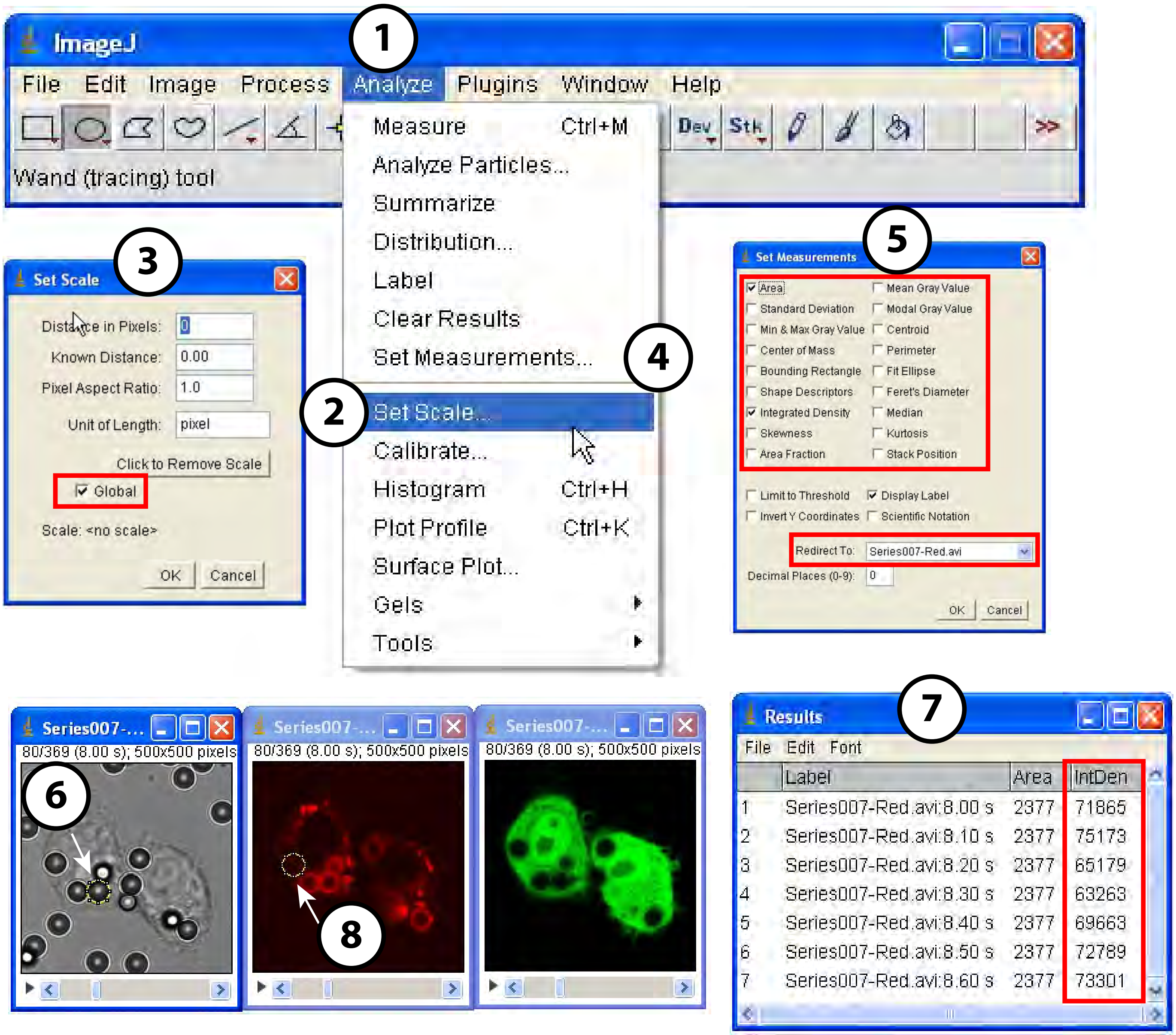

1
2
3
4
5
6
7
8
ImageJ
File Edit Image Process Analyze Plugins Window Help
Wand (tracing) tool
Measure Ctrl+M
Analyze Particles...
Summarize
Distribution...
Label
Clear Results
Set Measurements...
Set Scale...
Calibrate...
Histogram Ctrl+H
Plot Profile Ctrl+K
Surface Plot...
Gels
Tools
Set Scale
Distance in Pixels: 0
Known Distance: 0.00
Pixel Aspect Ratio: 1.0
Unit of Length: pixel
Click to Remove Scale
Global
Scale: <no scale>
OK Cancel
Set Measurements
Area
Mean Gray Value
Standard Deviation
Modal Gray Value
Min & Max Gray Value
Centroid
Center of Mass
Perimeter
Bounding Rectangle
Fit Ellipse
Shape Descriptors
Feret's Diameter
Integrated Density
Median
Skewness
Kurtosis
Area Fraction
Stack Position
Limit to Threshold
Display Label
Invert Y Coordinates
Scientific Notation
Redirect To: Series007-Red.avi
Decimal Places (0-9): 0
OK Cancel
Series007-...
80/369 (8.00 s); 500x500 pixels
Results
File Edit Font
Label Area IntDen
1 Series007-Red.avi:8.00 s 2377 71865
2 Series007-Red.avi:8.10 s 2377 75173
3 Series007-Red.avi:8.20 s 2377 65179
4 Series007-Red.avi:8.30 s 2377 63263
5 Series007-Red.avi:8.40 s 2377 69663
6 Series007-Red.avi:8.50 s 2377 72789
7 Series007-Red.avi:8.60 s 2377 73301

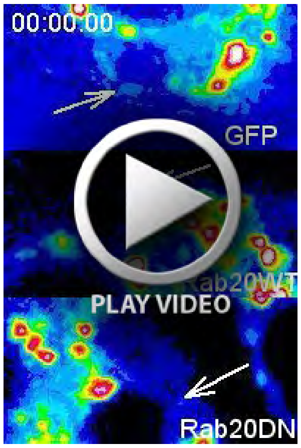

Movie 1.

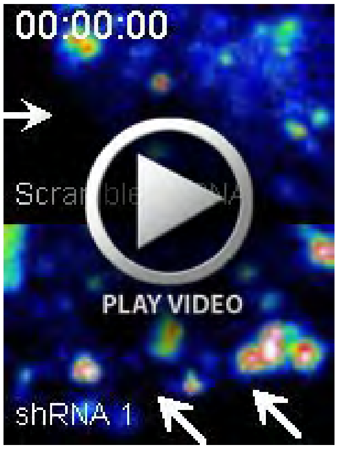

Movie 2.

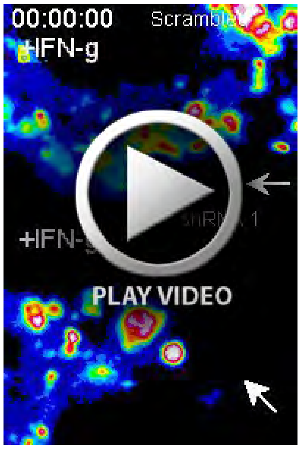

Movie 3.
